# Polarization Properties in Apertureless-Type Scanning Near-Field Optical Microscopy

**DOI:** 10.1186/s11671-015-1062-5

**Published:** 2015-09-29

**Authors:** Takayuki Ishibashi, Yongfu Cai

**Affiliations:** Department of Materials Science and Technology, Nagaoka University of Technology, 940-2188 Kamitomioka, Nagaoka, Niigta, Japan

**Keywords:** a-SNOM, Polarization property, FDTD, Jones matrix, 07.79.Fc, 42.25.Ja, 02.70.Bf

## Abstract

Polarization properties of apertureless-type scanning near-field optical microscopy (a-SNOM) were measured experimentally and were also analyzed using a finite-difference time-domain (FDTD) simulation. Our study reveals that the polarization properties in the a-SNOM are maintained and the a-SNOM works as a wave plate expressed by a Jones matrix. The measured signals obtained by the lock-in detection technique could be decomposed into signals scattered from near-field region and background signals reflected by tip and sample. Polarization images measured by a-SNOM with an angle resolution of 1° are shown. FDTD analysis also reveals the polarization properties of light in the area between a tip and a sample are p-polarization in most of cases.

## Background

Recently, nano-optics are attracting great attentions not only for measuring physical properties such as optical responses [1, 2], Raman scattering [3], and magnetic properties but also for reactions of molecules and/or controlling of spins. In those cases, polarization states in nano-optics are very important, because those phenomena are dependent on the polarization of light. For example, spin manipulation with light is attracting attentions because light may have abilities of controlling the direction of magnetization in a time scale of several tenth nanoseconds. Stanciu et al. reported that magnetization direction of GdFeCo is optically switched by a 40 fs circularly polarized light (CPL) pulse [4]. Satoh et al. demonstrated the spin-wave emission and the directional control of propagation using the CPL or linearly polarized light (LPL) [5]. These new phenomena may lead to controlling single spin with light. On the other hand, CPL has been also used to measure circular dichroism (CD) due to the geometric and electromagnetic chiral properties of single molecule [6], biomolecules [7], and singe-wall carbon nanotube [8]. In those cases, obviously, each single molecule can be selectively investigated if nano-sized CPL is available.

For high resolved polarization imaging with a spatial resolution of ~10 nm, scanning near-field optical microscopies (SNOM) have been developed in the 1990s [9–12]. Aperture-type SNOMs using optical probe having an aperture on top of tapered optical fiber with metal were extensively studied, because it was considered that polarization properties were maintained and background signal was relatively small. Magneto-optical (MO) measurements that measure optical responses of magnetic materials for circular polarization are one of the most important purpose at that time [11–22], because size of magnetic recording marks had became smaller than the wavelength of the visible light. Several groups use aperture-type optical fiber probes collecting the evanescent wave, which is referred to as a transmission-mode MO-SNOM. However, there are disadvantages. When the evanescent wave propagates through the optical probes, the light intensity decreases extremely, the polarization of the wave is deteriorated, and the spatial resolution of the imaging was limited to the size of the aperture, which is typically larger than 50 nm. In addition, it was difficult to measure opaque materials in reflection-mode, because the thickness of metallic coating contributes to enlargement of probe end.

Another type of SNOM, apertureless-type SNOM (a-SNOM), has also been developed, in which cantilever tips for atomic force microscopy were used instead of the optical fibers probe with an aperture. In a-SNOM, the scattered light generated at the area where evanescent waves are formed between the tip’s extremity and the sample’s surface. Considering the principle of a-SNOM, we can expect higher spatial resolution depending on the radius of tips, which is smaller than 10 nm in the commercially available tip, with higher intensity and good polarization properties. In addition, it is considered that a-SNOM is suitable for measurements of opaque materials, since there is no obstacle around the extremity of tips. One of the most successful applications of a-SNOM is tip-enhance Raman scattering spectroscopy [23–27]. On the other hand, polarization property of a-SNOM has not been sufficiently understood yet. In this paper, we report the polarization properties of a-SNOM, including experimental and simulation results.

## Methods

### a-SNOM setup

**Fig. 1:**
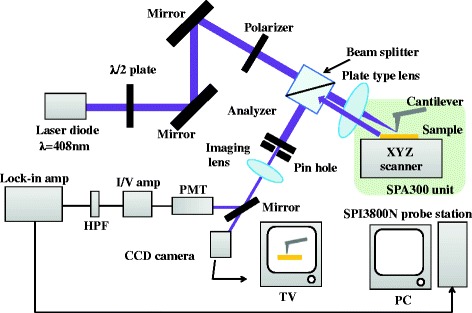
A schematic of a-SNOM developed in our study [28]

The a-SNOM setup is illustrated in Fig. 1. We used a commercial scanning probe microscopy (SPM) from Seiko Instrument Inc., model SPI3800N probe station and SPA300 unit, as a base instrument, and during the experiment process, we selected the dynamic force mode (DFM). The tip is SI-DF3P2 SII NanoTechnology Inc., made of silicon having an extremity’s radius of 7 nm and a resonant frequency *Ω* of 77 kHz. A laser beam (TC20-4030-4.5/15, NEOARK) with a wavelength of 408 nm propagates through a half-wave plate and a polarizer to ensuring a certain polarization direction, i.e., either *s*- or *p*-polarization direction, later is passing to a prism-type beam splitter and is finally focused at the tip apex by a plate-type lens. An angle between a sample surface and the incident beam is 45°. A scattered beam from the tip and the sample’s surface is collected by the same lens, reflected by the beam splitter, and is passing through an imaging lens. Signals were detected using a lock-in detection technique and demodulated at frequencies *Ω* or 2*Ω*.

### FDTD simulation

**Fig. 2:**
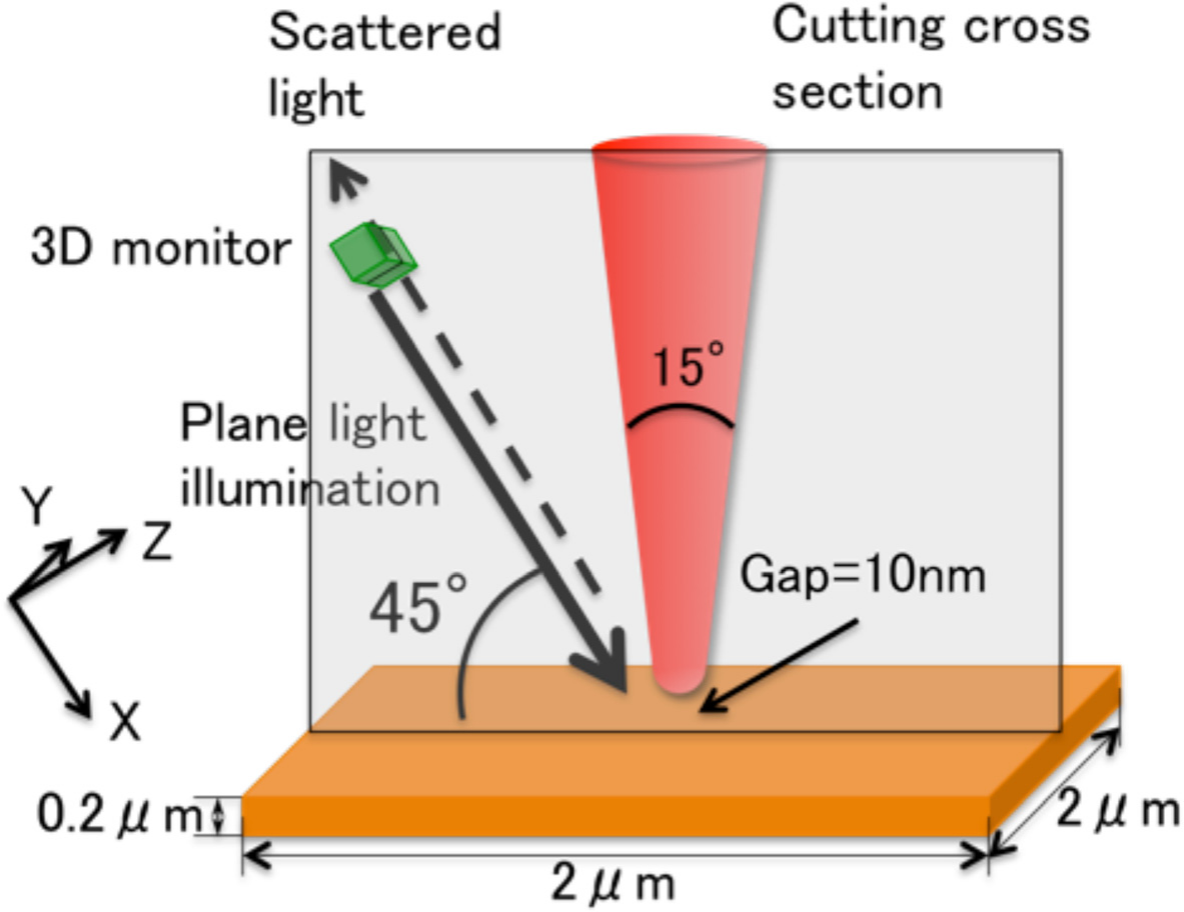
A model of a-SNOM used for our simulation [29]

The finite-difference time-domain (FDTD) method is a straightforward calculation of Maxwell’s equations in both time and space. In our simulation, we used a commercial soft, FullWAVE (RSoft Design Group, Inc.). Figure 2 shows a schematic drawing of a simulation model used in this study. A conical probe made of silicon (*n* = 5.476 + i0.310 at the wavelength of 408 nm) with an extremity’s radius of 7 nm and a solid angle of 15° was located above the Cr (*n* = 1.80 + i3.61 at the wavelength of 408 nm) film. A distance between the probe and the Cr was 10 nm. A pulse plane wave with a wavelength of 408 nm and a pulse width of 2.1 fs impinged on the tip and the sample with an incident angle of 45°. 3D monitors were located in front of light source to record the electric field of incident light and in the behind of light source to record the electric field of scattered light. The size of our model was 3 × 3 × 3 μm^3^, using 3 × 3 × 3 nm^3^ cell. The probe was set with normal to the Cr surface to study fundamental polarization properties of a-SNOM, although there was an inclination of approximately 10° in the actual experiment [29, 30].

**Fig. 3:**
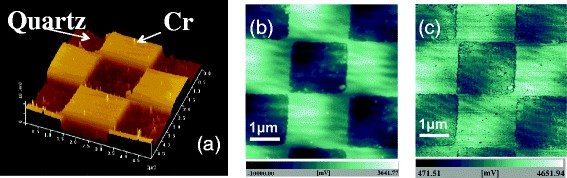
**a** An AFM image and **b**, **c** SNOM images of Cr pattern on quartz substrate. **b** and **c** were measured with demodulation frequency of *Ω* and 2*Ω*, respectively [28]

## Results and Discussion

We measured a chromium film deposited on a quartz substrate with a checker pattern with a thickness of 20 nm and a period of 2 × 2 μm^2^. Figure 3 shows a topographic image and two SNOM images of the chromium pattern measured with the *s*-polarized illumination [28]. Figure 3b, c was measured at demodulation frequencies of *Ω* and 2*Ω*, respectively. In those SNOM images, the checker pattern corresponding to the topography in Fig. 3a is clearly observed. We found that the intensity of area of chromium film (higher parts) is higher than that of quartz area (lower parts), and they showed opposite sign of phase *ϕ* for the chromium and the quartz. This result indicates that the signal depends on not only the reflectivity but also the phase. Therefore, we consider that the contrast obtained in those SNOM images is due to difference in the complex permittivity of the materials. In Fig. 3c, a dark area surrounding the chromium patterns is observed. We believe this is due to the edge darkening effect. Spatial resolution was determined to be 14 nm by measuring a cross section of SNOM images measured at 2*Ω*.

**Fig. 4:**
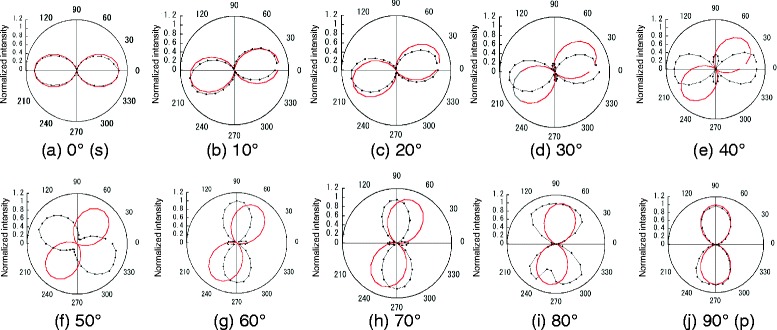
**a**–**j** Polar plots for SNOM intensities measured with various angles of analyzer. *Dots* are measured data, and *red solid lines* show linearly polarization expressed by cos^2^ *θ*

To analyze polarization properties of the a-SNOM, we measured the intensity of SNOM signal with an analyzer. Figure 4 shows polar plots of the intensities measured at demodulation frequency of 2*Ω* with linearly polarized incident lights with azimuth angles of 0° to 90° plotted as a function of the analyzer angles. Red solid lines show ideal linearly polarizations of the azimuth angles of the incident lights. In Fig. 4a, j, corresponding to s- and p-polarizations, respectively, the polarizations of measured data coincide with those of the incident light, suggesting the polarization of light was maintained. However, polarization properties of SNOM signals showed different properties except for s- and p-polarizations, as shown in Fig. 4b–i. For example, clover-shaped patterns were observed in some cases, Fig. 4d, e, g, and h, indicating that measured signals consisted of two or more components with different polarizations as described below.

**Fig. 5:**
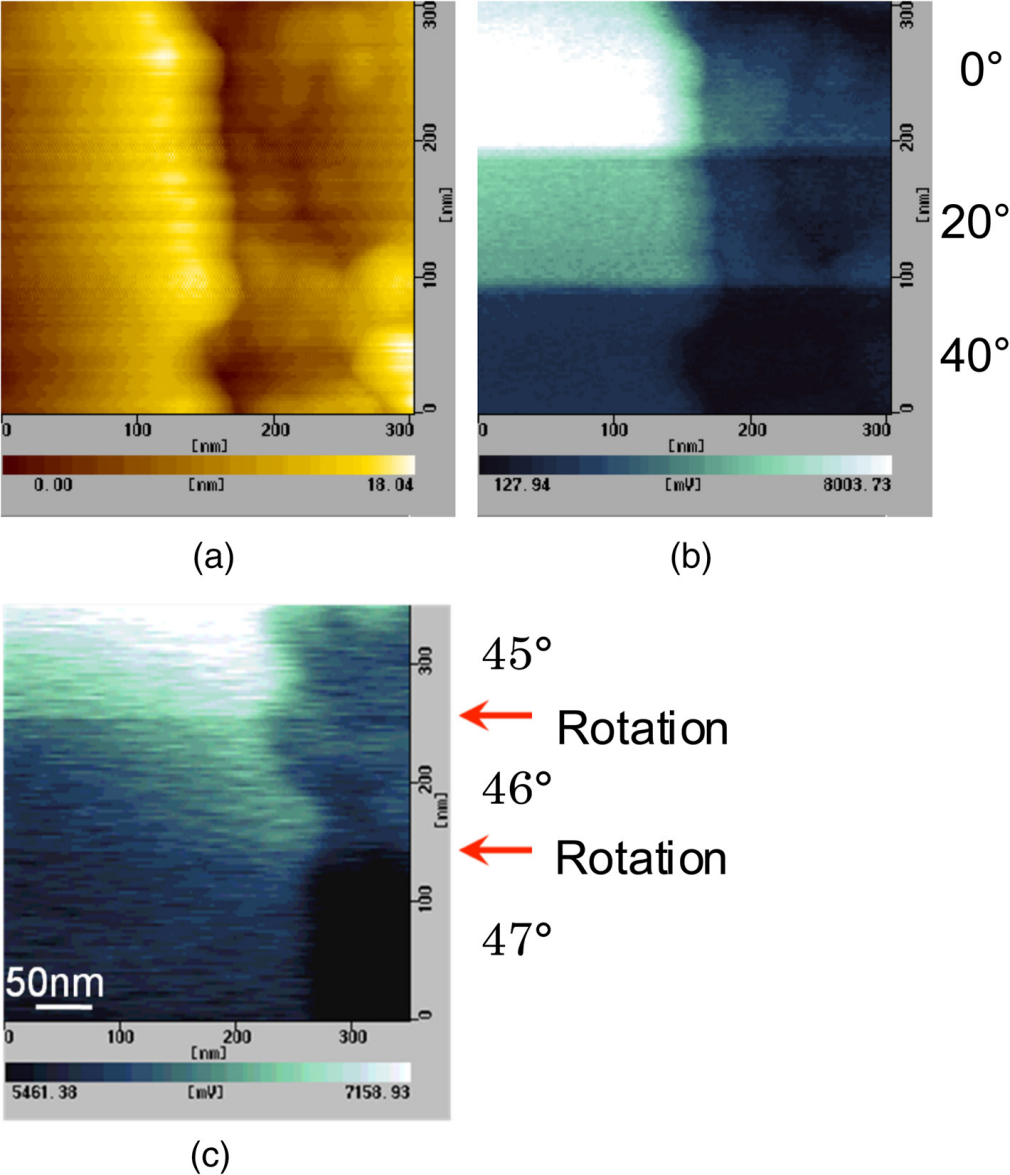
**a** An AFM image and **b**, **c** SNOM images of a Cr pattern on quart substrate measured with various angles of the analyzer shown in the *right side* of the images

Since the polarization seems to be maintained in the case of s- and p-polarization illuminations, we measured polarization SNOM images in order to confirm it. Figure 5 shows an AMF image and SNOM images of a Cr pattern on quart substrate measured with s-polarization illumination. Figure 5b was measured with the analyzer angles of 0°, 20°, and 40° as indicated on right side of the figure, where the angle was changed during the measurement. The intensity of the SNOM image was clearly decreased with the analyzer angle was increased, which corresponded to the result shown in Fig. 4a. Furthermore, we measured a SNOM image with the analyzer angles of 45°, 46°, and 47° as shown in Fig. 4c. As a result, the polarization images were successfully measured with the angle resolution better than 1°.

**Fig. 6:**
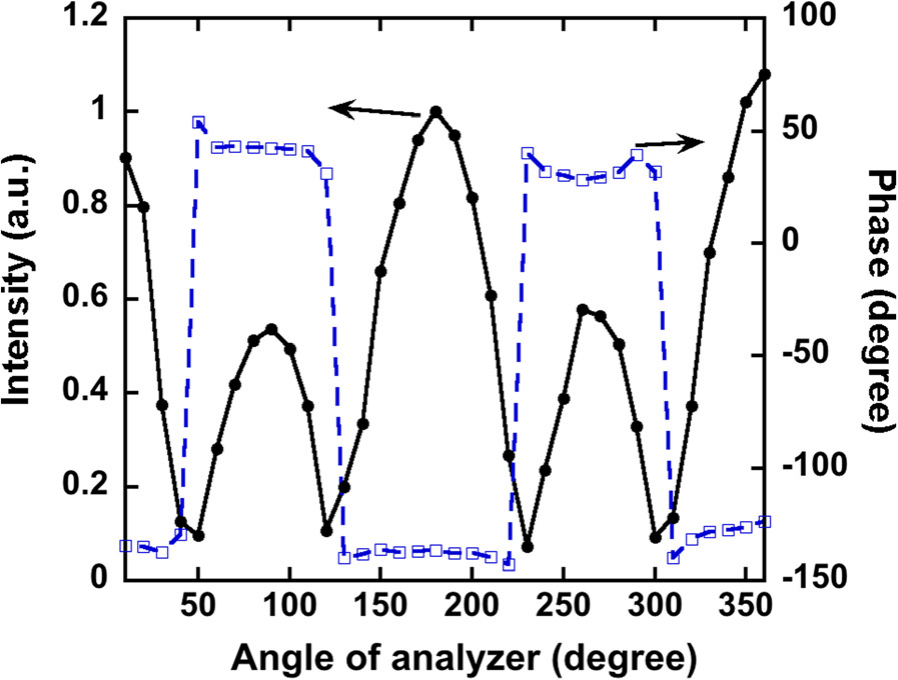
Intensity and phase of SNOM signal measured with the lock-in amplifier

**Fig. 7:**
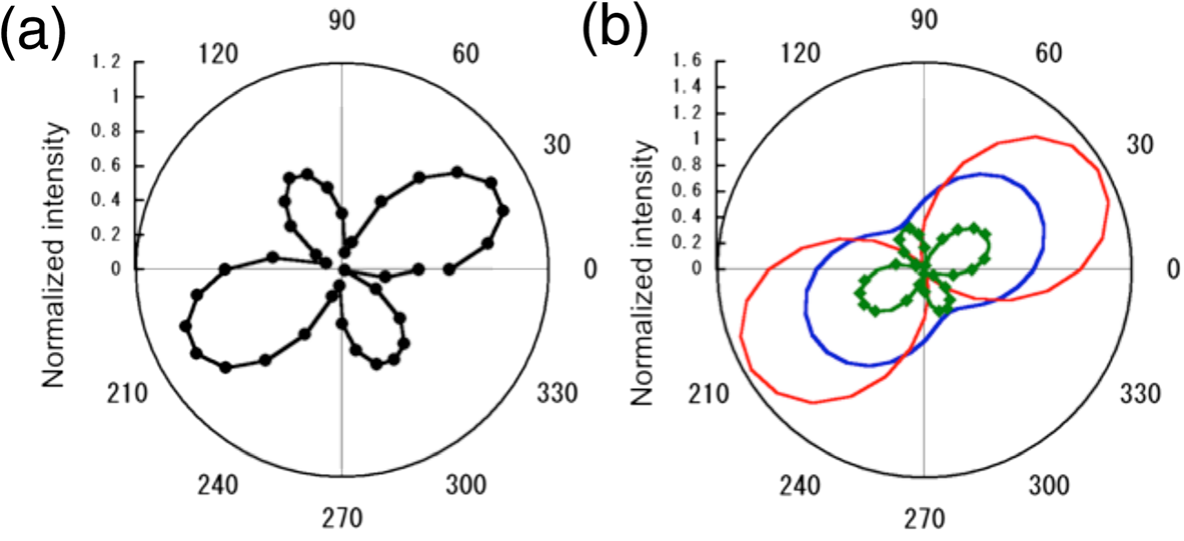
**a** A polarplot of SNOM signal plotted as a function of angle of analyzer and **b** a result of decomposition

**Fig. 8:**
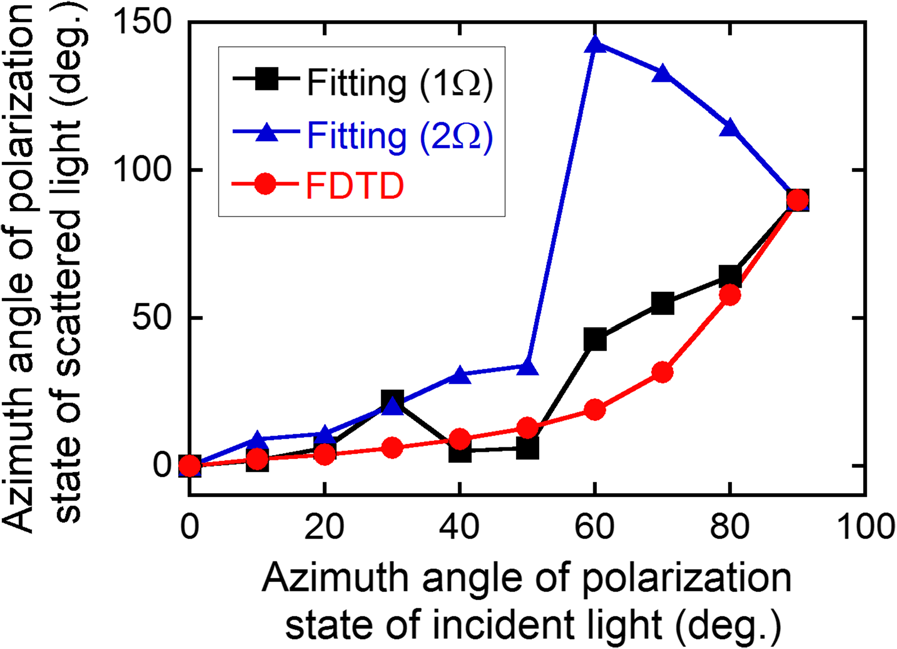
Azimuth angles of SNOM signals deduced by the fitting plotted as a function of azimuth angles of the incident light, together with a result of FDTD simulation

Although polarization images were successfully measured in Fig. 5, we have to mention that the measured signals consist of two or more polarized light. Figure 6 shows the intensity and the phase measured by the lock-in amplifier plotted as a function of the angle of the analyzer. The intensity changed with the angle of the analyzer and had peaks, corresponding to the clover-shaped pattern in Fig. 4. It is found that the phase suddenly changed and the angle difference was 180°, suggesting that there were at least two different signals involved. For example, considering SNOM signal has a maximum intensity when the tip approaches close to a sample surface in DFM mode, the other signal should have a maximum intensity when the tip separates from the sample surface. Therefore, we assume that measured signals consisted of two polarized lights, SNOM signal and background signal, described in an equation,

1 $$ I={\left|{E}_{\mathrm{N}}\right|}^2{ \cos}^2\left(\theta +{\psi}_{\mathrm{N}}\right)+{\beta}_{\mathrm{N}}+{\left|{E}_{\mathrm{B}}\right|}^2{ \cos}^2\left(\theta +{\psi}_{\mathrm{B}}\right)+{\beta}_{\mathrm{B}} $$

where *E* is strength of electric field, *θ* is the angle of the analyzer, *ψ* is the azimuth angle of the polarized light, *β* corresponds to ellipticity, and N and B denote SNOM signal from near-field region and background signal, respectively. In this analysis, we also assume the background signal was a light reflected by a tip made of Si and a sample made of Cr. As a consequence, we can obtain the parameter, *ψ*_B_ and *β*_B_, using optical constants of Si and Cr. Once the polarization of the background signal was obtained, we can determine the other parameters by fitting of measured data. An example of the fitting is shown in Fig. 7. The measured data shown in Fig. 7a can be decomposed into two polarized light, SNOM signal in blue and background signal in red, as shown in Fig. 7b. In all cases, SNOM signals were successfully deduced by the fitting procedure, and the results summarized in Fig. 8. show azimuth angles of SNOM signals measured for the azimuth angle of the incident light of 60° and demodulation frequencies *Ω* and 2*Ω* are plotted together with a result obtained by the FDTD simulation [30]. In the case of *Ω*, the azimuth angles of SNOM signals gradually increased with the angle of the azimuth of the incident light, which agreed with the result by FDTD. On the other hand, data for 2*Ω* agreed with FDTD for the angle below 50°, but it behaved differently for 60°–80°. We can not explain the reason of the difference, but it may be due to the shape of the apex of the tip, because the measurement at a demodulation frequency 2*Ω* has stronger distance dependence than that at *Ω*.

**Fig. 9:**
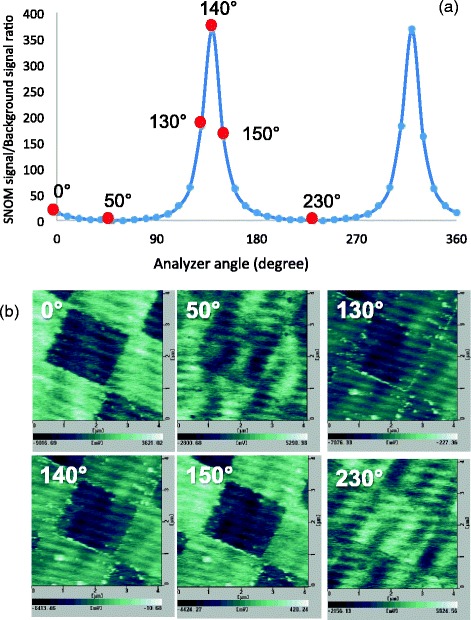
**a** Ratios of SNOM signals to background signals plotted as a function of the analyzer, and **b** SNOM images of a Cr pattern on quart substrate measured with various angles of analyzer indicated in (**a**), 0°, 50°, 130°, 140°, 150°, and 230°

The ratios of the SNOM signals to the background signals measured for the azimuth angles of the incident light of 60° were plotted as a function of the analyzer angles in Fig. 9a. It was found that there are peaks at 140° and 320°, which are quite different from the azimuth angles of the incident light of 60°. If this is true, SNOM could be measured with good S/N ratio at those peak positions. In order to prove the validity of our analysis, we measured SNOM images of the Cr pattern with various angles of analyzer, 0°, 50°, 130°, 140°, 150°, and 230° as shown in Fig. 9b. Clear SNOM image was obtained for 0° and 150°, which is close to the peak position in Fig. 9a. On the contrary, the SNOM images measured for 50° and 230°, close to the azimuth angle of the incident light, suffered from noise due to background signals. From these results, we can conclude that our analysis is valid.

From the FDTD simulation, we also found that the a-SNOM system can be described by Jones matrix as described [30],

2 $$ T=\left[\begin{array}{l}3.954\kern2.5em 0\\ {}\kern1em 0\kern1em  \exp \kern0.5em (0.710i)\end{array}\right] $$

**Fig. 10:**
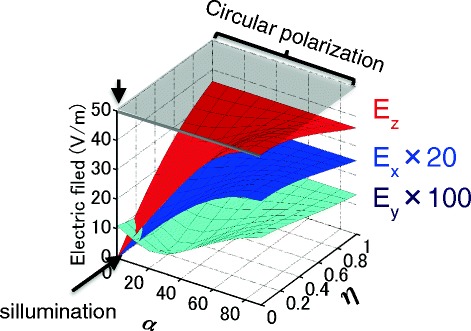
Electric filed components, *E*_x_, *E*_y_, and *E*_z_ are plotted as a function of the azimuth angles and the ellipticity of the incident light

This result indicates that a-SNOM maintains the polarization of light and works as a wave plate. However, we found that the polarization state in the near-field region between the tip and the sample is only in p-polarization except for the case that both azimuth angle and ellipticity of the incident light is almost zero. Figure 10 shows strengths of electric filed components, *E*_x_, *E*_y_, and *E*_z_, plotted as a function of the azimuth angles and the ellipticity of the incident light. In most of the cases, including the case of the circular polarization, strength of *E*_z_ was the largest, indicating that linear polarization is p-polarization. This result indicates the difficulty of measuring the optical responses due to circular polarization, such as CD and MO effect, by using a-SNOM, which is consistent with the theoretical prediction [31]. To overcome this problem, we need to consider another type of tip that can produce nano-sized circular polarized light [32].

## Conclusions

Polarization properties of apertureless-type scanning near-field optical microscope (a-SNOM) were studied. Polarization SNOM images were successfully measured with the spatial resolution of ~14 nm and the angle resolution better than 1°. We found that the polarization properties of optical signals could be decomposed into SNOM signal scattered from near-field region and background signal reflected by a tip and a sample. Signal to noise ratio was improved by choosing the angles of the incident light and the analyzer. We also found that a-SNOM worked as a wave plate described by Jones matrix and the polarization state of the electric field between a tip and a sample was p-polarization.
